# Analysis of distribution characteristics of COVID-19 in America based on space-time scan statistic

**DOI:** 10.3389/fpubh.2022.897784

**Published:** 2022-08-10

**Authors:** Yuexu Zhao, Qiwei Liu

**Affiliations:** College of Economics, Hangzhou Dianzi University, Hangzhou, China

**Keywords:** the spatiotemporal analysis, COVID-19, scan statistic, spatial aggregation, Omicron

## Abstract

Based on the epidemic data of COVID-19 in 50 states of the United States (the US) from December 2021 to January 2022, the spatial and temporal clustering characteristics of COVID-19 in the US are explored and analyzed. First, the retrospective spatiotemporal analysis is performed by using SaTScan 9.5, and 17 incidence areas are obtained. Second, the reliability of the results is tested by the circular distribution method in the time latitude and the clustering method in the spatial latitude, and it is confirmed that the retrospective spatiotemporal analysis accurately measures in time and reasonably divides regions according to the characteristics in space. Empirical results show that the first-level clustering area of the epidemic has six states with an average relative risk of 1.28 and the second-level clustering area includes 18 states with an average relative risk of 0.86. At present, the epidemic situation in the US continues to expand. It is necessary to do constructive work in epidemic prevention, reduce the impact of epidemic, and effectively control the spread of the epidemic.

## Introduction

Coronavirus disease 2019 (COVID-19) refers to the new coronavirus infection in 2019 caused by acute respiratory infectious diseases in the majority of patients; some of them will develop as severe cases and even results in death. Since the large-scale outbreak of new corona pneumonia in 21 January 2020, the economy in the US has been gradually affected. Besides, the epidemic is a public emergency that all countries in the world have to face. As one of the most serious epidemic countries, the US has accumulated 74,741,586 new coronavirus cases as on 31 January 2022, and there is a certain aggregation tendency in time and space. Scan statistic is a method to test whether there is an aggregation of diseases, and detect whether the abnormal increase of diseases in time and space is caused by random variation. It has been widely used in infectious diseases, cardiovascular diseases, and other fields as a spatial statistical method in epidemic statistics.

In 1965, Joseph (1) proposed the concept of scan statistic. Kulldorff et al. (2–5) proposed the spatial scan statistic, and applied scan statistic to analyze the breast cancer mortality in the US. For example, they utilized the dynamic variable scanning window to detect the leukemia data in northern New York, and used the log-likelihood ratio to determine the cluster with the highest degree of aggregation. They also proposed many statistical models of spatiotemporal scanning, such as retrospective space-time scan statistic in Bernoulli model or Poisson model, prospective space-time scan statistic, space-time rearrangement scan statistic, and elliptical spatial scan statistic in periodic geographic disease monitoring. As the research goes further, Jung et al. (6, 7) proposed ordinal model scan statistic in 2007, which had excellent performance compared with Bernoulli scan statistic for binary classification of prostate cancer data. Huang et al. (8, 9) proposed the spatial scan statistic based on the exponential model for the male survival data with prostate cancer in the US in 2007; this method could be applied to the survival data and pure spatial data. In order to study the spatial heterogeneity continuously measured in the population data, the weighted normal spatial scan statistic was proposed and applied to the two-stage lung cancer survival research in 2009. Barbara (10) found that Cutl's method was more effective than Kulldorff's scan statistic for irregular shape spatiotemporal clusters, and for cylindrical spatiotemporal clusters; these two methods had similar results. Li et al. (11) analyzed the fund sustainability. Yin (12) carried out the research on application in early warning of infectious diseases, and graded the data of provinces and cities. Ma et al. (13) selected the optimal spatial scale through the number of signals in the monitoring of infectious diseases. So far, scan statistic has been widely used in disease prevention, including tuberculosis, schistosomiasis, and hand, foot, and mouth disease.

The majority of the abovementioned studies explore the spatial aggregation of various infectious diseases. COVID-19 is a highly contagious disease, which has seriously affected people's lives since its outbreak, and has a great threat to people's health. Hohl et al. (14) used the daily new coronavirus case data provided by the John Hopkins University at the county level, and applied SaTScan to conduct a prospective space-time analysis, and detected the active clusters in various provinces and cities in the US. To avoid using prospective space-time scan statistic to identify emergence of COVID-19 disease groups, Beard et al. (15) proposed the COVID-19 monitoring method, which was based on spatiotemporal event sequence similarity. Hohl et al. (16) used prospective Poisson space-time scan statistic to detect daily clusters of COVID-19 at successive county levels in 48 states and Washington DC, which was helpful to facilitate decision-making and public health resource allocation. Pei et al. (17) found that the epidemic distribution had obvious space-time heterogeneity, and the spatial-temporal transmission had typical network characteristics.

In this paper, we will study the spatial aggregation of COVID-19 in the US from the following aspects. First, we construct a dynamic scanning window, calculate the relative risk to measure the intensity of aggregation, and utilize the scan statistical analysis through SaTScan9.5 based on the retrospective spatiotemporal analysis method. Second, we analyze the rational treatment of SaTScan9.5, and innovatively use circular distribution method (time latitude) and cluster analysis method (spatial latitude) to test the reliability of spatiotemporal scanning results. Through horizontal comparison, it is found that spatiotemporal scan analysis not only accurately measures in time but also reasonably divides regions according to characteristics in space. Finally, we take into account the data and how the COVID-19 pandemic changes on the ground, locating the gathering area and span period on time. At the same time, according to the scanning results, it not only provides an important theoretical basis for the relevant epidemic prevention work, but also has crucial importance for the establishment of an early warning system for the corresponding disease, ultimately playing a positive role in strengthening prevention and resolving the risk of major diseases in the world.

## Methodology

Retrospective spatiotemporal analysis needs to build a scanning window to judge the number of diseases inside and outside the window. Since the scanning statistics involve time and space, the scanning window is in the form of a cylinder; the height of the cylinder represents the time, and the bottom area of the cylinder represents the area. The location and size of the scan window are dynamic, as it is unknown when and where the COVID-19 outbreak will occur.

In the analysis process, a position is randomly selected as the scanning center, and then, the cylindrical scanning window changes continuously. The cluster of geographic size of the scanning window ranges between zero and a predefined upper limit. There are several ways to determine the value of upper bound, for example, one can take the percentage of number of people at risk of disease or radius value of circle as the upper bound. In this article, we use the former method. The time length of the scan window specifies the maximum time frame according to the percentage of the entire study cycle or the specific number of days.

To determine the possibility of aggregation, the actual number of patients and the number of regional populations are calculated to obtain the theoretical number of patients, and the log-likelihood ratio (*LLR*) is constructed by using the actual and theoretical number of patients inside and outside the window; the relative risk ($\tilde{R}$) is calculated to evaluate the strength of aggregation. Since the scanning window undergoes a dynamic change, numerous scanning windows will be generated during the scanning process. For controlling the false-positive rate at a certain level, the window with the largest *LLR* is selected as the clustering area among all scanning windows. The statistical significance of *LLR* is tested by Monte Carlo stochastic simulation method.

We then give hypothesis test as follows:

Null Hypothesis (*H*_0_): *The spatial and temporal distribution of newly confirmed cases of COVID-19 in the US is completely random;*

Alternative Hypothesis (*H*_1_): *The spatial and temporal distribution of newly confirmed cases of COVID-19 in the US is not completely random*.

Assuming that the number of cases in window *A* is *n*_*A*_, the population is *m*_*A*_, *E*(*A*) is the expected number of cases in the scanning window based on the original assumption and adjusted by covariates, the total number of cases in the total region is *n*_*T*_, the total population is *m*_*T*_, and the expected number of cases is *E*(*T*), then

(1) $$\begin{array}{r} E\left(A\right)=\frac{n_{I}}{m_{I}}\times m_{A} \end{array}$$

(2) $$\begin{array}{r} E\left(T\right)=\sum E\left(A\right) \end{array}$$

The probability density function of specific points observed at region *x* is as follows:

(3) $$\begin{array}{r} f\left(x\right)=\{\begin{array}{c} \frac{pE\left(x\right)}{pE\left(A\right)+q\left[E\left(T\right)-E\left(A\right)\right]},x\in A\\ \frac{qE\left(x\right)}{pE\left(A\right)+q\left[E\left(T\right)-E\left(A\right)\right]},x\notin A \end{array} \end{array}$$

where *p* is the ratio of actual incidence to expected incidence in window *A*, *q* is the ratio of actual incidence to expected incidence outside window *A*, and the probability of any specific point is independent of all other points, one can also refer to Tang et al. (18) and Yang (19).

If *p* > *q*, the likelihood function *LR*(*A, p, q*) is denoted by:

(4) $$\begin{array}{l} LR\left(A,p,q\right)=\\ \frac{e^{-n_{T}}}{n_{T}!}{\left(\frac{n_{A}}{E\left(A\right)}\right)}^{n_{A}}{\left(\frac{n_{T}-n_{A}}{E\left(T\right)-E\left(A\right)}\right)}^{n_{T}-n_{A}}\underset{x_{i}\in A}{\prod }E\left(x_{i}\right) \end{array}$$

Otherwise, the likelihood function *LR*_0_(based on invalid hypothesis) is

(5) $$\begin{array}{r} LR_{0}=\frac{e^{-n_{T}}}{n_{T}!}{\left(\frac{n_{T}}{E\left(T\right)}\right)}^{n_{A}}\underset{x_{i}\in A}{\prod }E\left(x_{i}\right) \end{array}$$

Test statistic for spatiotemporal scan λ is defined as follows:

(6) $$\begin{array}{r} \lambda :\;=\;\frac{\underset{A,p>q}{Sup}LR\left(A,p,q\right)}{\underset{A,p=q}{Sup}LR\left(A,p,q\right)} \end{array}$$

According to Equations (4) and (5), we have

(7) $$\begin{array}{r} \lambda =\underset{A}{Sup}{\left(\frac{n_{A}}{E\left(A\right)}\right)}^{n_{A}}\;{\left(\frac{n_{T}-n_{A}}{E\left(T\right)-E\left(A\right)}\right)}^{n_{T}-n_{A}}{\left(\frac{n_{T}}{E\left(T\right)}\right)}^{-n_{T}}I\left(B\right) \end{array}$$

(8) $$\begin{array}{r} B:=\left\{\frac{n_{A}}{E\left(A\right)}>\frac{n_{T}-n_{A}}{E\left(T\right)-E\left(A\right)}\right\} \end{array}$$

In formula (7), *I*(·) is a characteristic function. The ratio of the actual incidence to the expected incidence in window *A* is greater than the ratio of the actual incidence to the expected incidence outside window *A*. The is a measure of how risk within a cylinder differs from risk outside.

Next, we use Monte Carlo random method to simulate the *p* value of *LLR* to determine whether the aggregation is statistically significant. First, we simulate *c* random datasets, calculate the maximum *LLR* for each dataset, and rank it with the real *LLR* from big to small. If the real value rank is *R*, then we have

(9) $$\begin{array}{r} p=R{\left(c+1\right)}^{-1} \end{array}$$

If *p* < 0.05, we reject the original assumption. The relative risk of each aggregation is as follows:

(10) $$\begin{array}{r} \tilde{R}=\left(\frac{n_{A}}{E\left(A\right)}\right){\left(\frac{n_{T}-n_{A}}{E\left(T\right)-E\left(A\right)}\right)}^{-1} \end{array}$$

## Empirical analysis

### Source of the data

This paper selects 50 states from the US for research. The data of the COVID-19 mainly came from the data of the New York Times, including the date of diagnosis, the current area, and the source of infection of patients with COVID-19. Demographic data mainly came from the US 2020 census data, the basic geographic information data of each state were derived from Google satellite map data, and the latitude and longitude coordinates mainly chose the state capital as the center position.

The variant data are derived from the Centers for Disease Control and Prevention (CDC) study that tracks the proportion of variants estimated from weekly random sampling in the Department of Health and Human Services region followed by gene sequencing tests across the region, which we use to estimate the number of variant infections in the state over a week with new cases per day.

### Parameter setting

We use the retrospective spatiotemporal analysis method, and choose the discrete Poisson model. The scanning time is set to be from 1 December 2021 to 31 January 2022, and the time interval is 1 day. As COVID-19 is highly contagious, the population with a ceiling of 50% in the space window is at risk, and the maximum circle size file is set at 30% of the population, rather than 30% of the regular population, and the regional overlap is set at zero. Referring to a large number of relevant literatures, combined with the actual situation, it is known that the outbreak of COVID-19 is fast, and the incubation period is short. Besides, the inaction of the US government to manage the outbreak makes the cycle longer. The daily pattern of COVID-19 changes rapidly, so the minimum time cluster is set to 1 day. In the test window, the number of Monte Carlo random simulation is set to 999.

### Description analysis

**Figure 1:**
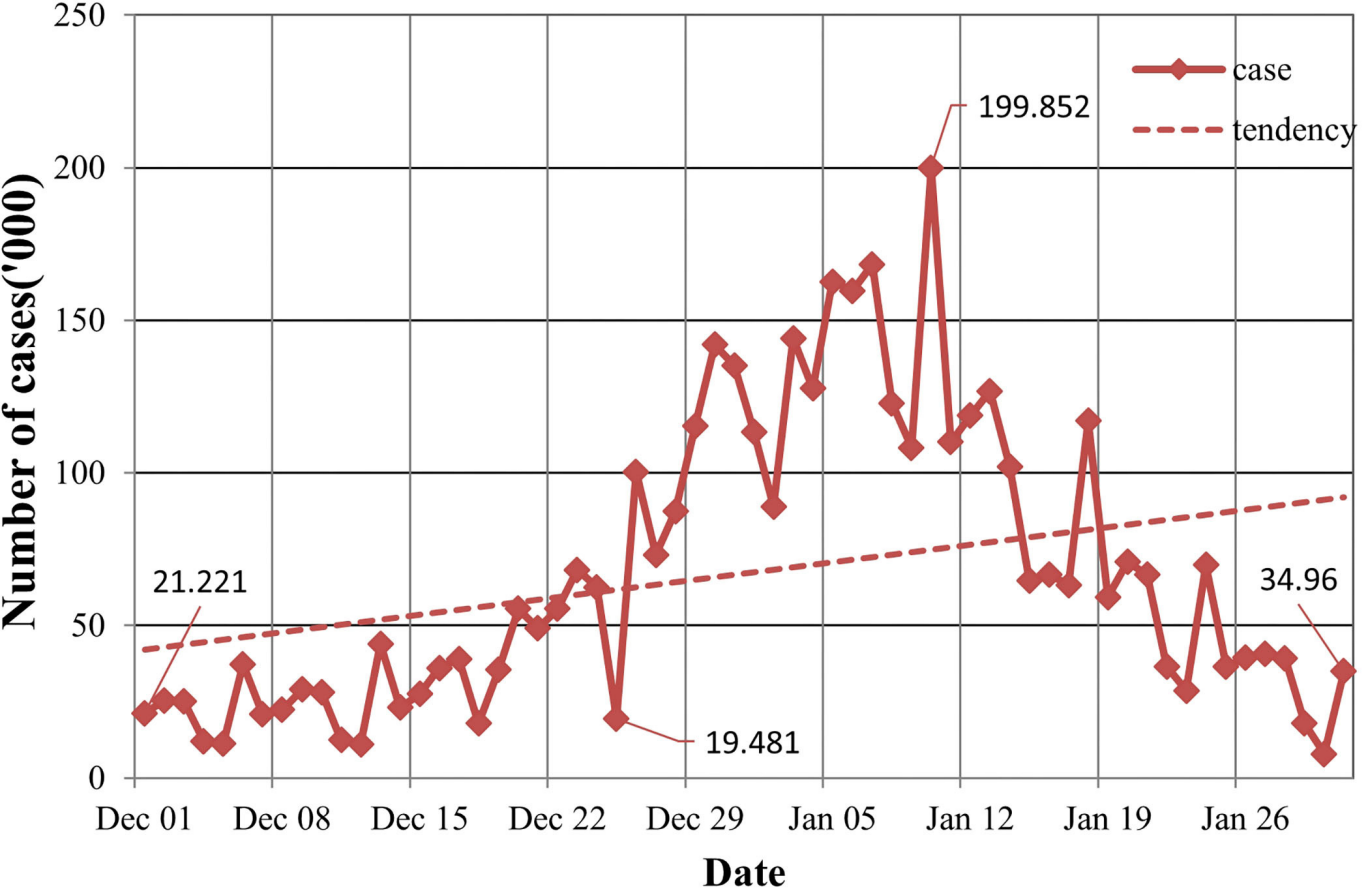
The number of COVID-19 cases.

In the population distribution, the US COVID-19 has nothing to do with gender, and included patients mainly in the age group of 44 to 59 years. In terms of time distribution, the US had the largest number of new cases on 10^th^ January, with 1,420,374 cases. On 3^rd^, 18^th^, and 24^th^ January, more than 1 million new cases were added daily with 1,003,751, 1,173,885, and 1,025,999 cases, respectively. In terms of the overall trend, the outbreak in the early stages of each state is relatively serious, and the number of confirmed cases has experienced a short lag and rapid growth. From Figure 1, we can see that the overall epidemic situation has not been effectively controlled, so the number of confirmed cases has increased cumulatively, having a certain increasing tendency. In the regional distribution, cases were mainly concentrated in the east and west of the US, and California has the largest number of confirmed cases, followed by New York.

### Space-time analysis

A retrospective spatiotemporal analysis is carried out in the US. After SaTScan 9.5 is run, 17 clustering areas are obtained and arranged from large to small according to the log-likelihood ratio, and we obtain *p* < 0.01. The clustering areas are tested by the aboriginality test. The specific data are summarized in Table 1.

**Table 1:** Retrospective spatiotemporal analysis.

**Cluster**	**Start date**	**The number of states**	**The actual value**	**Value of expectation**	** $\tilde{R}$ **	***LLR***	***p***
1	Dec 1	6	4,157,904	3,363,765.04	1.28	101,152.5	0.0000
2	Dec 1	18	5,817,663	6,526,216.68	0.86	52,598.68	0.0000
3	Dec 1	1	1,891,720	2,301,321.62	0.81	42,332.33	0.0000
4	Dec 1	5	1,114,238	1,418,944.71	0.78	37,219.81	0.0000
5	Dec 1	4	1,965,177	2,274,140.59	0.85	24,001.90	0.0000
6	Dec 1	2	945,695	1,169,284.65	0.80	23,886.30	0.0000
7	Dec 1	1	672,175	845,809.54	0.79	19,780.36	0.0000
8	Dec 1	6	882,793	1,034,449.98	0.85	12,161.49	0.0000
9	Dec 1	2	3,455,831	3,236,835.98	1.08	8,298.06	0.0000
10	Dec 1	1	1,844,383	1,700,649.87	1.09	6,333.58	0.0000
11	Dec 1	2	1,351,620	1,228,440.97	1.11	6,284.42	0.0000
12	Dec 1	1	535,773	465,366.48	1.15	5,172.76	0.0000
13	Dec 1	1	1,110,569	1,011,672.39	1.10	4,878.70	0.0000
14	Dec 1	1	423,426	485,990.37	0.87	4,288.31	0.0000
15	Dec 1	3	1,056,440	990,205.76	1.07	2,254.76	0.0000
16	Dec 1	3	2,193,108	2,263,168.50	0.97	1,198.58	0.0000
17	Dec 1	1	381,903	355,779.67	1.07	949.66	0.0000

The cluster areas are mainly concentrated in Connecticut, Rhode Island, New York, Massachusetts, New Hampshire, and New Jersey from 1 December 2021 to 31 January 2022. The log-likelihood ratio is 101,152.56, and the aggregation is the highest, with a relative risk of 1.28. It also shows that the aggregation of COVID-19 in the six places during this period is strong. From 1 December 2021 to 31 January 2022, 18 states, such as Colorado, become the second agglomeration, with a log-likelihood ratio of 52,598.68 and a relative risk of 0.86. Texas from 1 December 2021 to 31 January 2022 is one of the three types of gathering areas, with a log-likelihood ratio of 42,332.33 and a relative risk of 0.81.

Combined with the daily incidence of each state, it can be observed that the starting time of the gathering area is just the time for the sudden increase of the confirmed cases of COVID-19 in the region, and the end time is the time for the growth rate of the confirmed cases to begin to decline. Combined with Table 2, the incidence of the four states involved, Rhode Island, New York, Massachusetts, and New Jersey, accounts for the top five regions of the incidence of COVID-19 in the US, and New York is the city with the second largest number of confirmed cases. Although Massachusetts and New Hampshire have fewer confirmed cases than New York, they are geographically close to New York, where the epidemic is relatively serious.

**Table 2:** Numbers of cases and morbidities in the top 20 states.

**State**	**Population**	**Case**	**Morbidity**	**Rank**
Rhode Island	1,097,379	152,016	0.138526434	1
New York	20,201,249	2,060,920	0.102019435	2
Massachusetts	7,029,917	692,497	0.098507137	3
Delaware	989,948	94,921	0.095884834	4
New Jersey	9,288,994	859,151	0.092491286	5
South Carolina	5,118,425	466,698	0.091180002	6
Wisconsin	5,893,718	535,773	0.090905775	7
Kansas	2,937,880	264,696	0.090097621	8
Alaska	733,391	65,639	0.089500689	9
Hawaii	1,455,271	129,489	0.088979304	10
Utah	3,271,616	289,753	0.088565712	11
Illinois	12,812,508	1,110,569	0.086678502	12
Louisiana	4,657,757	399,318	0.085731823	13
Florida	21,538,187	1,844,383	0.085633159	14
North Carolina	10,439,388	884,922	0.084767613	15
Kentucky	4,505,836	381,903	0.084757412	16
West Virginia	1,793,716	151,977	0.08472746	17
Vermont	643,077	54,458	0.084683483	18
California	39,538,223	3,326,342	0.08412978	19
Arizona	7,151,502	600,864	0.084019273	20

### Circular distribution analysis

Since the research time is 62 days, we divide 360° evenly over each day, then 1 day is equivalent to 5.81°, and 1 h is equivalent to 0.21°. To avoid the infinite calculation, the calculation time of each day is 8:00 a.m., that is, the one-third corresponding degree of 1 day is taken as the degree of the day. By the spatiotemporal scanning analysis, we obtain 17 clustering areas, and take the first three clustering areas as example. In order to compare the following analysis results to previous ones, we combine the daily newly confirmed cases according to the clusters. The *r*, *r*_0_, and *p*-values of Rayleigh test are obtained through calculation, as summarized in Table 3.

**Table 3:** Results of circular distribution analysis.

**Cluster**	***r***	***r*_0_**	***p***
1	0.4392	0.0013	0.001
2	0.4769	0.0011	0.000
3	0.5398	0.0019	0.001

The peak day and peaks of each cluster are summarized in Table 4.

**Table 4:** Peak day and peak period of incidence of COVID-19 in each cluster area.

**Cluster**	** $\bar{\alpha }$ **	***s***	**Peak incidence**	**Epidemic peak period**	**Peak period span (Day)**
1	32.7887	73.5005	Dec 6	Dec 6–Dec19	14
2	87.1093	69.7240	Dec 16	Dec 4–Dec28	25
3	77.1640	63.6210	Dec 14	Dec 3–Dec24	12

### Clustering analysis

**Figure 2:**
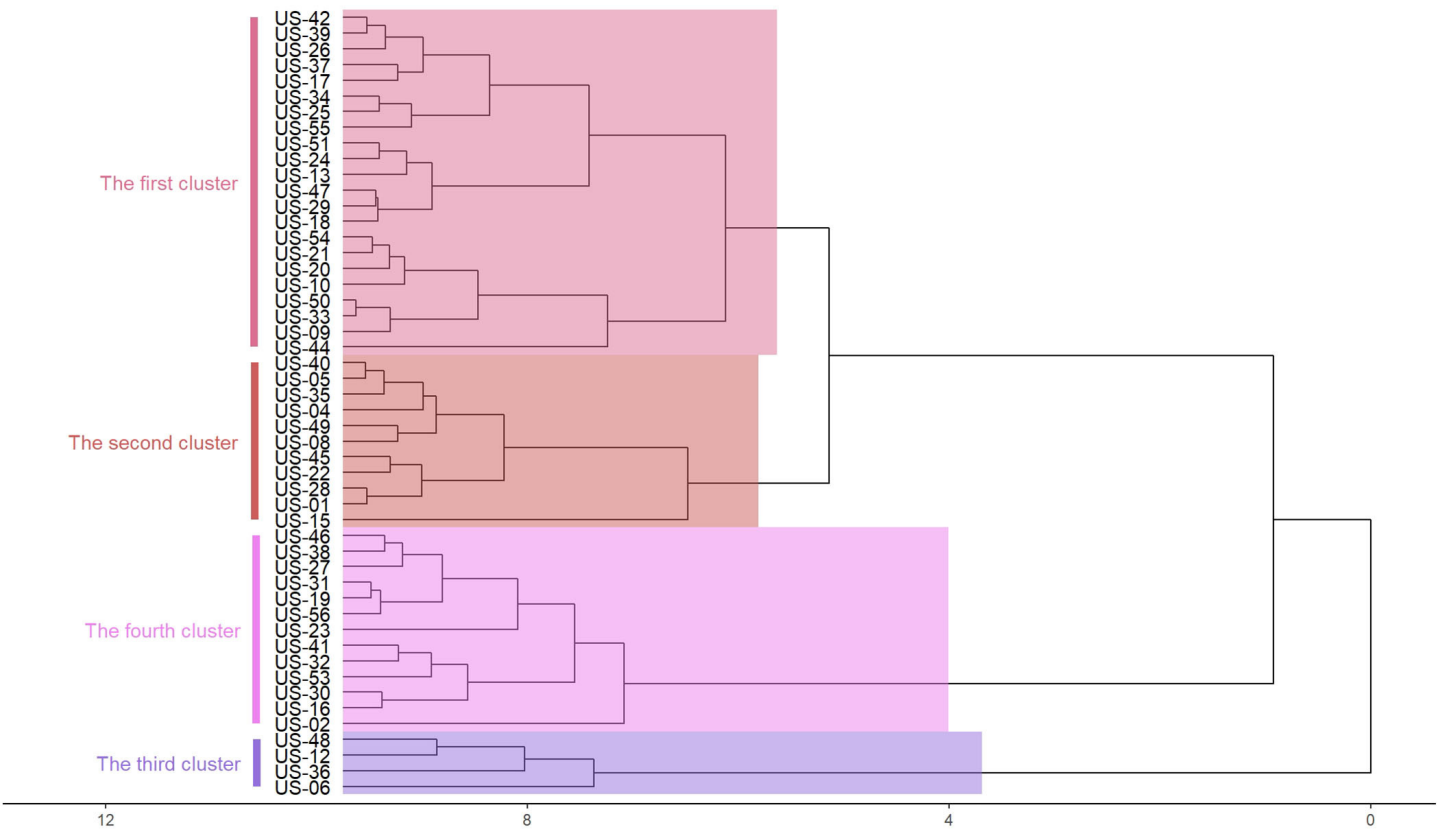
Coordinated hierarchical cluster analysis.

Hierarchical cluster analysis method is commonly used in classification research. This method can overcome the shortcomings of qualitative classification. According to the index characteristics of the classification object, the total feature similarity is divided into a class. In this case, the cumulative confirmed cases, the regional population, and the incidence rate are used as the indicators of each region, and imported into R software for standardization. The deviation square and clustering analysis are used to divide them into four categories. Since the latitude and longitude coordinates are involved in the spatiotemporal scanning analysis, the central coordinates of the capital are added to the index, as shown in Figure 2. Due to the mess up of text and pictures as displayed in the diagram, it should be replaced with a geographical code (US-01), as shown in Table 5.

**Table 5:** Correspondence tables of states.

**Code**	**State**	**Code**	**State**
US-01	Alabama	US-30	Montana
US-02	Alaska	US-31	Nebraska
US-04	Arizona	US-32	Nevada
US-05	Arkansas	US-33	New Hampshire
US-06	California	US-34	New Jersey
US-08	Colorado	US-35	New Mexico
US-09	Connecticut	US-36	New York
US-10	Delaware	US-37	North Carolina
US-12	Florida	US-38	North Dakota
US-13	Georgia	US-39	Ohio
US-15	Hawaii	US-40	Oklahoma
US-16	Idaho	US-41	Oregon
US-17	Illinois	US-42	Pennsylvania
US-18	Indiana	US-44	Rhode Island
US-19	Iowa	US-45	South Carolina
US-20	Kansas	US-46	South Dakota
US-21	Kentucky	US-47	Tennessee
US-22	Louisiana	US-48	Texas
US-23	Maine	US-49	Utah
US-24	Maryland	US-50	Vermont
US-25	Massachusetts	US-51	Virginia
US-26	Michigan	US-53	Washington
US-27	Minnesota	US-54	West Virginia
US-28	Mississippi	US-55	Wisconsin
US-29	Missouri	US-56	Wyoming

From Figure 2, we can see that there are highly correlated with geographical location. The same category of states are adjacent states, and the case information is not reflected. Therefore, the clustering method cannot well-balance the relationship between the number of cases and their geographical locations.

### Results comparison

The peak periods calculated by spatial-temporal scanning analysis are compared with those calculated by circular distribution method, as shown in Table 6. Combined with the actual situation, the peak period of the disease obtained by the circular distribution method is similar to the epidemic situation of COVID-19 in the region. The peak period of the disease obtained by the spatial-temporal scanning method is longer than that of the circular distribution method, and the time is generally advanced. Spatiotemporal scanning analysis can send early warning signals for COVID-19, which is a kind of fulminant and fast infectious disease, and has a higher practical value for disease prevention and control.

**Table 6:** Comparison of STSA and CDA.

**Cluster**	**Fastigium (STSA)**	**Span (Day)**	**Fastigium (CDA)**	**Span (Day)**
1	Dec 1–Jan 31	62	Dec 6–Dec 19	14
2	Dec 1–Jan 31	62	Dec 4–Dec 28	25
3	Dec 1–Jan 31	62	Dec 3–Dec 24	12

The clustering areas obtained by spatiotemporal scanning analysis are compared with the classification results obtained by the system clustering method, as shown in Table 7. In the Spatiotemporal Scanning Analysis (STSA), Hierarchical Cluster Analysis (HCA), and Coordinated Hierarchical Cluster Analysis (CHCA), US-44 (Rhode Island), US-25 (Massachusetts), US-09 (Connecticut), US-34 (New Jersey), and US-33 (New Hampshire) are classified into the first category, while US-36 (New York) is classified into the first category by spatiotemporal scanning. Combined with the actual situation, it can be seen that the results have a great relationship with the cumulative confirmed cases. After adding the coordinate index, the results are highly correlated with the geographical location, and the case information is weakened. The spatiotemporal scanning method makes good use of the information of regional population, case information, geographical location, and other information to give a reasonable clustering area. In terms of disease prevention and control, spatiotemporal scanning method can better provide theoretical basis for its adaptation to local conditions.

**Table 7:** Comparison of STSA and (C)HCA.

**Cluster**	**State (STSA)**	**State (HCA)**	**State (CHCA)**
1	US-09 US-44 US-36 US-25 US-33 US-34	US-15 US-02 US-10 US-54 US-33 US-50 US-38 US-35 US-28 US-40 US-09 US-05 US-21 US-22 US-55 US-20 US-45 US-49 US-04 US-34 US-25 US-44	US-09 US-10 US-13 US-17 US-18 US-20 US-21 US-24 US-25 US-26 US-29 US-33 US-34 US-37 US-39 US-42 US-44 US-47 US-50 US-51 US-54 US-55
2	US-08 US-56 US-35 US-49 US-46 US-31 US-20 US-40 US-38 US-04 US-30 US-19 US-16 US-29 US-27 US-48 US-05 US-32	US-08 US-01 US-27 US-29 US-47 US-18 US-53 US-51 US-13 US-37 US-26 US-17 US-42 US-39	US-01 US-04 US-05 US-08 US-15 US-22 US-28 US-35 US-40 US-45 US-49
3	US-48	US-36 US-12 US-48 US-06	US-12 US-36 US-06 US-48
4	US-53 US-41 US-16 US-30 US-32	US-32 US-19 US-41 US-24 US-46 US-31 US-56 US-23 US-16 US-30	US-02 US-16 US-19 US-23 US-27 US-30 US-31 US-32 US-38 US-41 US-46 US-53 US-56

Through the abovementioned comparative analysis, it can be seen that the circular distribution method and the space-time scan method have a certain overlap interval in the peak period of disease onset, and the clustering analysis method is certainly similar with its regional aggregation. However, the spatiotemporal scanning method can provide early warning and make better use of geographical factors to determine disease outbreak areas in detail, which is more instructive for the early warning and prevention and control of COVID-19.

The spatiotemporal scanning method can provide more objective grouping basis for the further model establishment of related research. According to the epidemic law of different regions, different groups can be included in different covariate modeling. The qualitative and quantitative research on the influencing factors of COVID-19 will provide an important basis for the development of effective epidemic prevention measures by health institutions such as disease control centers in the region by analyzing the incidence characteristics of patients with COVID-19 in different regions and at different times, and combining the economic level, population flow, medical conditions, and other factors in the region.

### Omicron variation

In the study of infectious diseases, we cannot ignore the situation of some variants. Based on the time node selected in this paper, the first Omicron case was reported in the US on 1 December, so we are paying attention to Omicron at this stage. Next, we need to know more about Omicron. In fact, Omicron has a significant growth advantage over Delta, leading to rapid spread in the community with higher levels of incidence than previously seen in this pandemic. With the sharp increase of cases and the scarcity of medical resources, we should also give importance to its dissemination.

**Figure 3:**
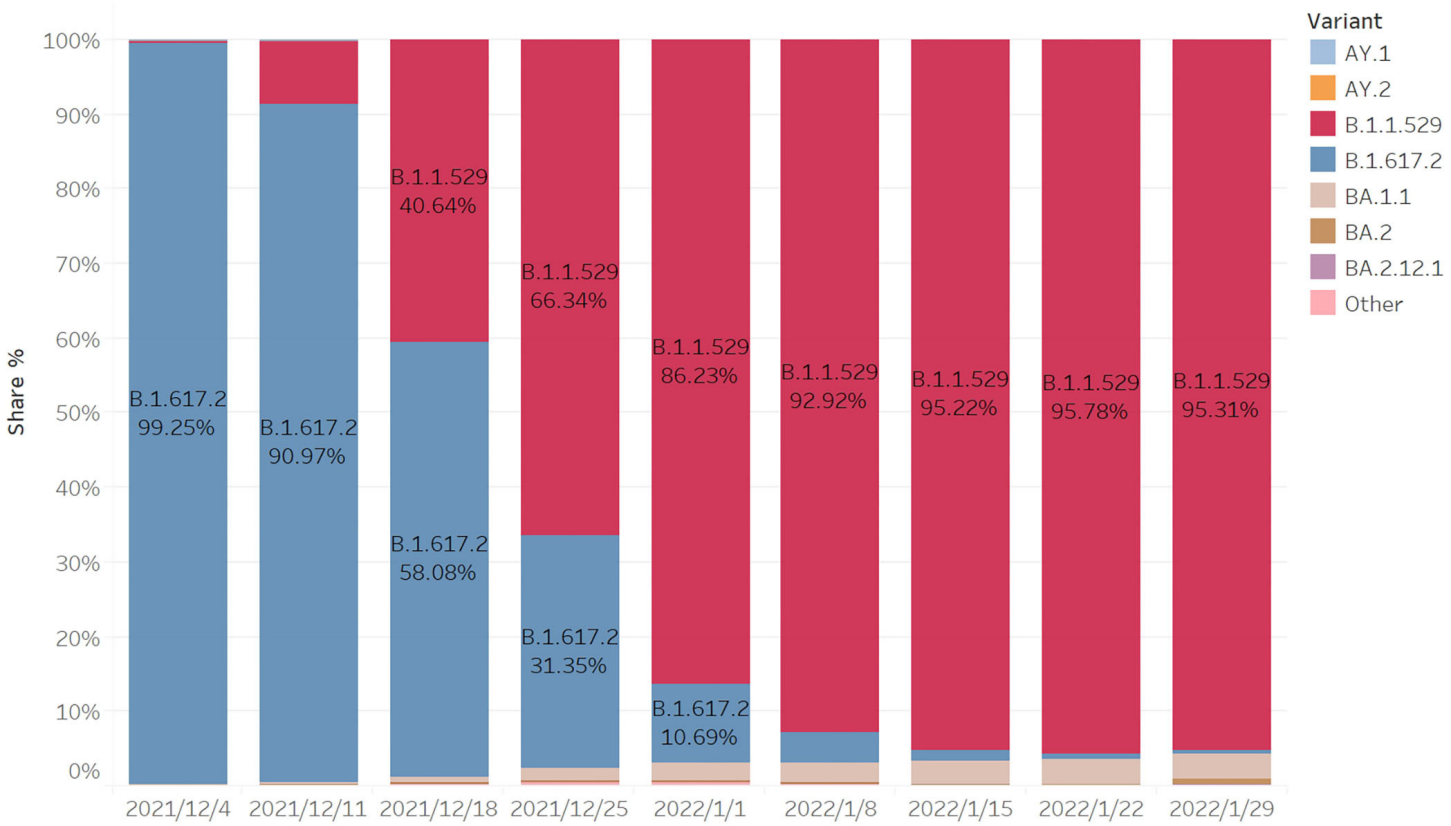
Proportion of COVID-19 variants.

From Figure 3, we can see that the B.167.2 (Delta) accounted for 99.25% on 4 December, while B.1.1.529 (Omicron) accounted for a low proportion. After 2 weeks, the proportion of Omicron increased rapidly, reaching 40.64%, while the corresponding Delta decreased to 58.08%. After another week, the proportion of Omicron exceeded Delta, becoming the largest variant of infection. After the following 5 weeks, the proportion reached 95.31%. Within 2 months, Omicron became the mutant with the largest proportion of infection, and its propagation speed was very fast.

**Figure 4:**
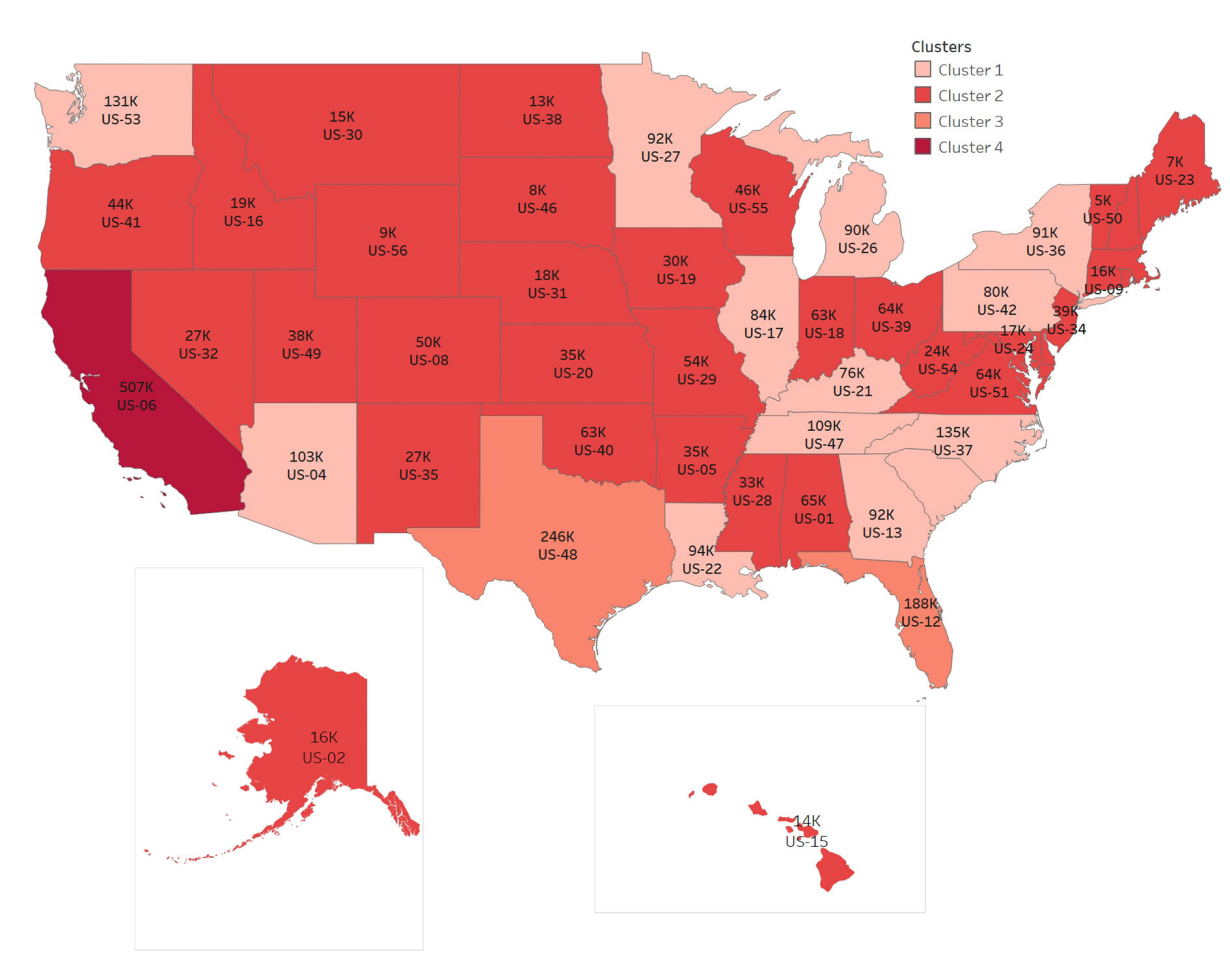
Distribution of Omicron cases from Jan 25 to Jan 31, 2022.

Based on the CDC's tracking data of variants and the prediction of the proportion of variants, we calculate the number of variants per week in different regions according to the new cases per day and the proportion of variants per week in the corresponding region. The following figure clearly shows the cumulative number of Omicron cases in the last week. In order to show the map integrally, US-02 and US-15 have changed the actual location in the map. According to the number clustering, the map is divided into four categories. We can find the features in Figure 4.

The numbers of items in clusters 1–4 are 18, 29, 2, 1, respectively, and the cluster centers are 89,703, 24,703, 217,010, 507,330 respectively, in Figure 4. The number of Omicron cases increases rapidly in 2 months, with the largest number of cases in one state, US-06, accumulating to 507,330 per week, and also with the largest number in neighboring states.

To summarize, it can be clearly seen that the rate of infection of Omicron increased rapidly, and there is a trend of diffusion from the middle to the surrounding. Population flow is one of the reasons for the rapid spread of virus. The reality of the spread from densely populated cities to other cities can also be observed from the distribution map of Omicron cases.

## Conclusion

In this paper, a retrospective spatiotemporal analysis of confirmed cases of COVID-19 in 50 states of the US is carried out. The first cluster is Connecticut, Rhode Island, New York, Massachusetts, New Hampshire, and New Jersey. The second cluster comprises 18 states, and the three types of gathering area is Texas. Through observation, it can be seen that the geographical location of the capital belonging to the same type of gathering area is relatively close. There is minimal difference between the gathering time and the peak time of newly confirmed cases daily, and the incidence of prominent gathering areas is higher. The reliability test of space-time scan results show that space-time scan has the advantages of accurate measurement in time and reasonable division of regions according to characteristics in space. On the basis of making full use of the existing time and spatial information, a spatiotemporal scanning analysis accurately locates the clustering area, timing and quantifying the corresponding clustering area, and evaluating the risk degree of the region, as we know that a high level of economic development and perfect medical conditions have played a positive role in the recovery of patients. From the analysis of this paper, spatiotemporal scanning analysis has greatly improved the timeliness and effectiveness of early warning of diseases, and can provide scientific basis for early prevention and control of diseases.

## Data availability statement

The original contributions presented in the study are included in the article/supplementary material, further inquiries can be directed to the corresponding author.

## Author contributions

YZ conceived and designed the study. QL analyzed the data. YZ and QL contributed to the writing of the manuscript. All authors contributed to the article and approved the submitted version.

## Conflict of interest

The authors declare that the research was conducted in the absence of any commercial or financial relationships that could be construed as a potential conflict of interest.

## Publisher's note

All claims expressed in this article are solely those of the authors and do not necessarily represent those of their affiliated organizations, or those of the publisher, the editors and the reviewers. Any product that may be evaluated in this article, or claim that may be made by its manufacturer, is not guaranteed or endorsed by the publisher.
